# The impact of development on patterns of nutrient limitation

**DOI:** 10.1111/1365-2435.13101

**Published:** 2018-04-16

**Authors:** Romain Richard, André M. de Roos

**Affiliations:** ^1^ Institute for Biodiversity and Ecosystem Dynamics University of Amsterdam Amsterdam The Netherlands

**Keywords:** co‐limitation, *Daphnia*, dynamic energy budgets, ecological stoichiometry, food quality, life history, threshold elemental ratios

## Abstract

1. Development is often accompanied by major changes in an organism's functioning and in the way it interacts with its environment. We consider how developmental events such as allocation changes at maturity, ontogenetic diet shift or metamorphosis may affect the likelihood and nature of nutrient limitation and explore the consequences of these changes in nutrient limitation for individual life history and patterns of biomass production.
2. To this purpose, we develop a general model for individual growth and reproduction that is based on the assumption that biomass production and metabolism require several nutrients and that individuals may require them in different proportion at different stages of their lives.
3. We parameterize this model for *Daphnia* based on its physiological requirements for carbon (C) and phosphorus (P). Growth and reproduction have different nutrient requirements, and this affects the likelihood of C vs. P limitation of differently sized individuals. This translates into a size‐dependent threshold elemental ratio (TER), with a difference of up to twofold between juveniles and adults, a difference comparable to measured interspecific differences.
4. The main implications of these findings are that, at the population level, co‐limitation of biomass production by several nutrients is likely to occur under a wide range of food qualities. In addition, different regimes of nutrient limitation strongly influence the relative difference in biomass production of differently sized individuals, which has been shown to be a major driver of population and community dynamics. Our results point to development as a key determinant of a population's response to food quality.

A http://onlinelibrary.wiley.com/doi/10.1111/1365-2435.13101/suppinfo is available for this article.

## 1 INTRODUCTION

Organisms require various nutrients to produce new biomass through somatic growth or reproduction. Nutrient availability is, however, often very variable over time and in space. Biomass production may therefore be limited by different substances depending on the environmental context. Ecological stoichiometry aims to integrate these effects and characterize their ecological consequences (Hall, 2009; Sterner & Elser, 2002). This approach has led to various insights in a range of fields from molecular biology to ecosystem functioning. A rather underappreciated, yet exciting, reason why it may be important to consider the possibility of multiple nutrient limitations is because nutrient requirements may vary throughout an individual's life (Moe et al., 2005; Simpson & Raubenheimer, 2011; Sterner & Schulz, 1998). All organisms grow during their life, and they may require different nutrients at different stages of development. A number of studies have tackled this question experimentally (e.g. Becker & Boersma, 2003; Simpson & Raubenheimer, 1993, 1997; Sterner & Schulz, 1998; Stockhoff, 1993; Urabe & Sterner, 2001), but overall, it remains largely unknown how development affects patterns of nutrient limitation and what are the ecological and evolutionary implications. In this study, we aim to stress the fundamental role of development in mediating patterns of nutrient limitation and present a general modelling framework that can be used to tackle these questions.

A great deal of effort has been invested to identify which properties of individual organisms determine the most‐limiting nutrient in given environments (Anderson, Hessen, Elser, & Urabe, 2005; Elser et al., 2000). The most frequently considered predictor is the body composition of the organism, which is then compared to that of its food. For example, we may expect that a species with a nitrogen‐rich body becomes nitrogen limited if its food is poor in nitrogen. Body composition is however generally not sufficient to provide accurate predictions, and many studies stressed the importance of considering basic physiological processes related to nutrient uptake and use, such as assimilation efficiencies, metabolic maintenance needs and overhead costs of biomass production (Anderson et al., 2005; Frost, Evans‐White, Finkel, Jensen, & Matzek, 2005). Many models have been developed to account for individual metabolism (e.g. Anderson & Hessen, 2005; Anderson et al., 2005; Shimizu & Urabe, 2008; Sterner & Elser, 2002), but none of the aforementioned studies accounts for the fact that an individual develops and that the importance of processes like metabolic maintenance needs and overhead costs for biomass production may vary throughout ontogeny. As far as we are aware, the only framework available that accounts comprehensively for both development and individual metabolism is the theory of multivariate dynamic energy budgets developed by Kooijman (2010, chapter 5). These models are however substantially complex, and there is a need for simpler models that are easier to understand, easier to parameterize from empirical data and easier to raise to supra‐individual levels.

The problem of ignoring development can be reinforced with the following example. Daphnids are certainly the type of organism that has been the most frequently used to study the consequences of variation in food quality on biomass production. However, almost every study that has tackled these types of questions did so by looking at how *juveniles* respond to food quality (e.g. Acharya, Kyle, & Elser, 2004; Elser et al., 2016; Hood & Sterner, 2014). Further implications for *Daphnia* biology and ecology are then drawn assuming that every individual within a population behaves like a juvenile. Adults may however respond very differently to variation in food quality (Urabe & Sterner, 2001). More generally, population‐ and community‐level conclusions are drawn assuming that every individual in the population responds in the same way to varying conditions of nutrient availability.

Identifying the most‐limiting nutrient requires a comparison of nutrient availability relative to the individual's actual needs (Moe et al., 2005). There are a number of logical reasons, supported through observations, to believe that both nutrient availability and nutrient requirements often vary throughout development. First, individuals may produce tissues of different compositions at different stages of their life. The most prominent example of such a change is that juvenile development implies the production of somatic tissues, whereas adult reproduction may require different nutrients to produce eggs. In support of this inference, differences in body and egg composition have been reported for many species (e.g. Færøvig & Hessen, 2003; Sterner & Schulz, 1998; Ventura & Catalan, 2005; Visanuvimol & Bertram, 2010). Furthermore, body composition itself often varies throughout development. Many studies have reported rather smooth changes in body composition with size (DeMott, Gulati, & Siewertsen, 1998; Frost & Elser, 2002; He & Wang, 2008) whereas very pronounced changes may also happen for organisms that metamorphose (Boros, Sály, & Vanni, 2015; Pilati & Vanni, 2007; Tiegs, Berven, Carmack, & Capps, 2016; Villar‐Argaiz, Medina‐Sanchez, & Carrillo, 2002). For example, copepods exhibit a series of metamorphoses throughout development, with drastic effects on their C:N:P composition (Villar‐Argaiz et al., 2002). Finally, an individual may also exhibit ontogenetic diet shifts (Werner & Gilliam, 1984). As the organism switches to a different food source, it is easy to imagine that it may become limited by another nutrient. The change here is external to the organism, but the source of that change is ultimately developmental, as it constitutes the trigger of the diet shift.

The importance of considering how multiple nutrient requirements interact with development can also be stressed from a completely different perspective. Development is indeed a fundamental process driving the dynamics of populations with numerous implications for higher levels of organization and evolution (De Roos & Persson, 2013). Approaches tackling these types of issues usually model the life history of individuals assuming that a single currency—usually energy—limits life‐history processes (e.g. De Roos & Persson, 2013; Economo, Kerkhoff, & Enquist, 2005; McCauley, Nelson, & Nisbet, 2008). As previously stressed, individuals in different states of development may be limited by different types of nutrients, and this is likely to affect how these individuals influence population‐level processes.

All these observations underline the need for an explicit account of development in stoichiometric models of biomass production and to account for the possibility of various types of nutrient limitation in life‐history models. Here, we aim to develop a simple and general model that would be the multiple nutrient equivalent of commonly encountered energetic models in the literature, such as net‐production and net‐assimilation models (e.g. Lika & Nisbet, 2000; Nisbet, McCauley, Gurney, Murdoch, & Wood, 2004; De Roos & Persson, 2013; Jager, Martin, & Zimmer, 2013). We then illustrate the implications of integrating ontogeny into a stoichiometric model (and vice versa) by parameterizing the model for *Daphnia* and exploring its behaviour. Finally, as we make the claim that our modelling framework is simple, we feel it is necessary to warn the reader that its derivation includes a few delicate steps, but the final model in itself is mathematically simple and is simple to interpret.

## 2 MATERIALS AND METHODS

In this section, we present a general model for the growth and reproduction of an individual under (potentially) multiple nutrient limitations. We specify the model in a way that does not rely on the specific details of the basic physiological functions, such as the assimilation, maintenance and allocation functions, allowing for flexibility and generality. In this model, biomass production requires both carbon *C* and another nutrient *X*, which we refer to as the mineral. Beside intrinsic differences in the dynamics of these two nutrients, a major difference is that we assume that individual body mass is directly proportional to its carbon content. This is a reasonable and rather frequently used assumption, as carbon usually makes some 40%–50% of an organism's dry wt (Sterner & Elser, 2002). Similarly, we assume that any other aspects (length, surface, volume, etc.) of an organism's size can be related to its body mass, and hence to its carbon content. Therefore, any size‐dependent rate can be expressed as a function of the individual's carbon content.

We aim here to derive expressions for the dynamics of body mass *W* and the reproductive output *R* of an individual throughout its life. Reproductive output is here meant as the production of neonate biomass. We assume that body composition is constant throughout ontogeny, with a C:X ratio denoted by ϕ_*B*_. This assumption is here made to avoid extra complexity in the derivations, but the framework can readily be extended to variable body composition, as explained in Discussion. As a consequence of this assumption, body growth and neonate production can be expressed using one nutrient only and we choose here to specify everything in carbon units. The quantity of the other nutrient fixed in biomass is implicitly defined by the requirement that all the biomass produced has a constant composition.

In what follows, we first describe the model in the case where carbon is the limiting nutrient. Next, we express model dynamics when the mineral is limiting and delineate the conditions under which the different types of nutrient limitation apply.

### 2.1 Dynamics under carbon limitation

The individual feeds on a food that has a concentration *F*, given in carbon units. Food ingestion rate is a function of both food concentration and individual size: *I*(*F*,* W*). A constant fraction σ_*C*_ of the ingested carbon is assimilated by the individual. Some carbon is used to fuel the metabolic maintenance needs linked to cell turnover and other energetic costs. This rate is related to individual mass and is given by *M*_*C*_ (*W*).

The production of new biomass comes at a cost, which may, for example, be associated with the energy required to produce new tissues. The associated overhead carbon costs of growth and reproduction are accounted for by the parameters γ_*CG*_ and γ_*CR*_, respectively. These parameters represent the fractions of carbon allocated to growth or reproduction that become actually sequestered in the newly produced soma. As we do not model explicitly embryogenic growth, γ_*CR*_ accounts for both the overhead cost for producing an egg and the provisioning of egg with energy to ensure embryogenesis.

At any age, a fraction θ(*F*,* W*) of the carbon assimilated in excess to maintenance is used for growth. We denote the difference between the assimilation and the maintenance rate as ω_*C*_ (*F*,* W*) = σ_*C*_ *I*(*F*,* W*) − *M*_*C*_ (*W*), so that:

(1) $$\frac{dW}{da}=\gamma_{\mathrm{CG}}\;\theta \left(F,\;W\right)\;\omega_{C}\left(F,\;W\right)$$

In many circumstances, one can assume that a juvenile allocates all carbon available to growth, in which case θ(*F*,* W*) = 1. Juveniles mature into adults once they reach a certain mass *W*_*j*_. It is also possible to use alternative schemes for the maturation process, which we address more extensively in Discussion section. In the adult stage, the remaining fraction of ω_*C*_ (*F*,* W*) is used for reproduction. The production rate of neonate biomass is given by:

(2) $$\frac{dR}{da}=\gamma_{\mathrm{CR}}\;\left(1-\theta \left(F,\;W\right)\right)\;\omega_{C}\left(F,\;W\right)$$

**Figure 1:**
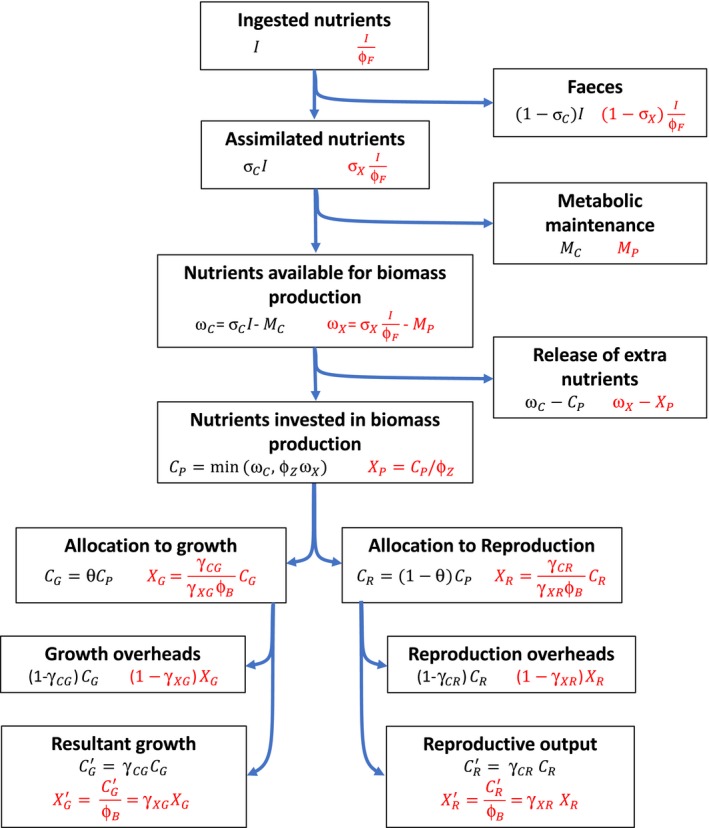
Partitioning of nutrients implied by the model. All quantities in boxes are rates. Their expressions are given in black for carbon dynamics and in red for mineral dynamics. To avoid notational burden, we did not include functions’ arguments

This model results in the channelling scheme depicted in Figure 1, although we warn the reader that the mathematical notation used in this figure may still be unclear, as further notation is introduced below to deal with multiple nutrient limitations.

We made θ a function of food concentration and the weight of the individual, so that it is possible to relate this model to many of the models previously published in the literature, as, when only carbon is limiting, it may collapse to a net‐production model or a net‐assimilation model. If θ = 1 in the juvenile stages and θ has a constant value, smaller than 1, in the adult stages, the model becomes a net‐production model. If $\theta \left(F,W\right)=\frac{\kappa \sigma_{C}I\left(F,W\right)-M_{C}\left(W\right)}{\omega_{C}\left(F,W\right)}$, with κ, the fraction of the assimilation rate allocated to growth, it becomes a net‐assimilation model.

### 2.2 Multiple nutrient model

Food composition is characterized by its C:X ratio, ϕ_*F*_. Therefore, the rate at which the mineral is ingested from food is $\frac{I(F,W)}{\phi_{F}}$. A constant fraction σ_*X*_ of the ingested mineral is assimilated by the individual, and some mineral is used for metabolic maintenance at a rate *M*_*X*_ (*W*). There are also potential overhead costs associated with the production of biomass through growth or reproduction reflected by the parameters γ_*XG*_ and γ_*XR*_, respectively. The difference between the assimilation and the maintenance rate of the mineral is $\omega_{X}\left(F,\;W,\;\phi_{F}\right)=\sigma_{X}\frac{I(F,\;W)}{\phi_{F}}-M_{X}\left(W\right)$.

As noted earlier, *W* and *R* are specified in carbon units, so that it becomes necessary to express how the dynamics under mineral limitation translates into carbon dynamics, which is more complex than it may appear at first sight. To this end, we introduce the functions *C*_*G*_ (*F*,* W*, ϕ_*F*_) and *X*_*G*_ (*F*,* W*, ϕ_*F*_), and the functions *C*_*R*_ (*F*,* W*, ϕ_*F*_) and *X*_*R*_ (*F*,* W*, ϕ_*F*_) as the quantities of each nutrient invested in somatic growth and reproduction, respectively. The sum of these nutrient investments *C*_*P*_ (*F*,* W*, ϕ_*F*_) = *C*_*G*_(*F*,* W*, ϕ_*F*_) + *C*_*R*_(*F*,* W*, ϕ_*F*_) and *X*_*P*_ (*F*,* W*, ϕ_*F*_) = *X*_*G*_ (*F*,* W*, ϕ_*F*_) + *X*_*R*_ (*F*,* W*, ϕ_*F*_) represents the quantities of each nutrient invested in overall biomass production. Importantly, however, we explicitly distinguish the resultants of these investments $C_{P}^{\prime }\left(F,W,\phi_{F}\right)$ and $X_{P}^{\prime }\left(F,W,\phi_{F}\right)$ (and similarly $C_{G}^{\prime }\left(F,W,\phi_{F}\right)$, $X_{G}^{\prime }\left(F,W,\phi_{F}\right)$, $C_{R}^{\prime }\left(F,W,\phi_{F}\right)$ and $X_{R}^{\prime }\left(F,W,\phi_{F}\right)$), which are the actual quantities of carbon and mineral, respectively, that end up sequestered in new biomass. It is formally not necessary to account for all these variables to get the full dynamics of the model, but it greatly clarifies the reasoning.

A key point here is that the relationships between the investment and resultant variables do not change under different scenarios of nutrient limitation. Therefore, we can eventually express all of these variables as a function of *C*_*P*_ (*F*,* W*, ϕ_*F*_). Different scenarios of nutrient limitation will affect the actual value taken by *C*_*P*_ (*F*,* W*, ϕ_*F*_), but, once again, not the relationship between the variables.

In the reasoning that follows, we first express the values of all investment and resultant variables as a function of *C*_*P*_ (*F*,* W*, ϕ_*F*_). We then use these results to infer the actual value of *C*_*P*_ (*F*,* W*, ϕ_*F*_) under mineral limitation. This produces the general formulation of the model shown in Table 1 and illustrated in Figure 1. We advise the reader to frequently return to Figure 1 while reading the derivations below, as it greatly helps to follow the logic of the model. Also, we directly present the derivations for the dynamics of adults, as juveniles dynamics are readily deduced from these equations by recognizing that no reproduction occurs. To ease the reading, we drop variable dependency in the derivations below.

**Table 1:** Model

*Balance equations*	
$\frac{dW}{da}=\gamma_{\mathrm{CG}}\;\theta \left(F,W\right)\;C_{P}\left(F,W,\phi_{F}\right)$	Growth
$\frac{dR}{da}=\left\{\begin{array}{cc} 0 & W<W_{j}\\ \gamma_{\mathrm{CR}}\left(1-\theta \left(F,W\right)\right)\;C_{P}\left(F,W,\phi_{F}\right) & W\geq W_{j} \end{array}\right.$	Reproduction
*Auxiliary functions (See text for definitions)*	
$C_{P}\left(F,\;W,\;\phi_{F}\right)=\min\left(\omega_{C}\left(F,\;W\right),\phi_{Z}\left(F,\;W\right)\;\omega_{X}\left(F,\;W,\;\phi_{F}\right)\right)$	
ω_*C*_ (*F*,* W*) = σ_*C*_ *I* (*F*,* W*) − *M*_*C*_ (*W*)	
$\omega_{X}\left(F,\;W,\;\phi_{F}\right)=\sigma_{X}\frac{I(F,W)}{\phi_{F}}-M_{X}\left(W\right)$	
$\phi_{Z}\left(F,\;W\right)=\frac{\phi_{B}}{\theta \left(F,\;W\right)\frac{\gamma_{\mathrm{CG}}}{\gamma_{\mathrm{XG}}}+\left(1-\theta \left(F,\;W\right)\right)\frac{\gamma_{\mathrm{CR}}}{\gamma_{\mathrm{XR}}}}$	
*Definitions of the primary functions and their specification for Daphnia*	
$I\left(F,W\right)=\frac{F}{F+F_{h}}\nu W$	Ingestion rate
*M*_*C*_(*W*) = *m*_*C*_ *W*	Maintenance rate for carbon
$M_{X}\left(W\right)=m_{X}\frac{W}{\phi_{B}}$	Maintenance rate for phosphorus
$\theta \left(F,W\right)=\left\{\begin{array}{cc} 1 & W<W_{j}\\ \frac{1}{1+r(W-W_{j})} & W\geq W_{j} \end{array}\right.$	Allocation function

Reexpressing carbon dynamics using the new notation, we get:

(3) $$C_{G}=\theta C_{P}$$

(4) $$C_{R}=\left(1-\theta \right)C_{P}$$

And,

(5) $$C_{G}^{\prime }=\gamma_{\mathrm{CG}}\theta C_{P}$$

(6) $$C_{R}^{\prime }=\gamma_{\mathrm{CR}}\left(1-\theta \right)C_{P}$$

In order to express the dynamics of the mineral as a function of *C*_*P*_, we now need to work backwards. We first start by considering that the biomass produced obeys stoichiometric constraints. This allows to get the expressions for rates at which the mineral is fixed in new biomass, $X_{G}^{\prime }$ and $X_{R}^{\prime }$, and further, to infer the original quantities of the mineral invested in these processes, *X*_*G*_ and *X*_*R*_.

As both somatic and neonate tissues have a constant composition ϕ_*B*_, we get:

(7) $$X_{G}^{\prime }=\frac{C_{G}^{\prime }}{\phi_{B}}$$

(8) $$X_{R}^{\prime }=\frac{C_{R}^{\prime }}{\phi_{B}}$$

Replacing $C_{G}^{\prime }$ and $C_{R}^{\prime }$ with the expression given in Equations (5) and (6):

(9) $$X_{G}^{\prime }=\frac{\gamma_{\mathrm{CG}}}{\phi_{B}}\theta C_{P}$$

(10) $$X_{R}^{\prime }=\frac{\gamma_{\mathrm{CR}}}{\phi_{B}}\left(1-\theta \right)C_{P}$$

Finally, accounting for the mineral overhead costs of biomass production:

(11) $$X_{G}=\frac{\gamma_{\mathrm{CG}}}{\phi_{B}\gamma_{\mathrm{PG}}}\theta C_{P}$$

(12) $$X_{R}=\frac{\gamma_{\mathrm{CR}}}{\phi_{B}\gamma_{\mathrm{PR}}}\left(1-\theta \right)C_{P}$$

when *C* is limiting, all the carbon available is used, which means that *C*_*P*_ = ω_*C*_, and Equations (9), (10), (11), (12) can be readily assessed. When *X* is limiting, all the mineral available is used so that *X*_*P*_ = *X*_*G*_ + *X*_*R*_ = ω_*X*_. Using the expressions for *X*_*G*_ and *X*_*R*_ above, we can rearrange this equality to get an expression for *C*_*P*_ under mineral limitation:

(13) $$C_{P}=\frac{\phi_{B}}{\theta \frac{\gamma_{\mathrm{CG}}}{\gamma_{\mathrm{XG}}}+\left(1-\theta \right)\frac{\gamma_{\mathrm{CR}}}{\gamma_{\mathrm{XR}}}}\omega_{X}$$

We remind here that ω_*X*_ is a known quantity derived from the assimilation and maintenance rates, which therefore only depends on the body mass *W*, food density *F* and food quality ϕ_*F*_. It is also useful to identify the quantity $\frac{\phi_{B}}{\theta \frac{\gamma_{\mathrm{CG}}}{\gamma_{\mathrm{XG}}}+\left(1-\theta \right)\frac{\gamma_{\mathrm{CR}}}{\gamma_{\mathrm{XR}}}}$ as the composition of the flux of nutrients invested in biomass production. We denote this quantity by ϕ_*Z*_ (*F*,* W*) and note that it is a function of both *F* and *W* because of the dependence of θ on these two variables.

To summarize, we have C_P_ = ω_C_ (*F*,* W*) under carbon limitation, and $C_{P}=\phi_{Z}\left(F,\;W\right)\;\omega_{X}\left(F,\;W,\;\phi_{F}\right)$ under mineral limitation. To identify the type of nutrient limitation, we now simply need to identify the type of nutrient that limits biomass production the most, in other words, to identify which one of the two previous expressions results in the smallest value of *C*_*P*_. The general expression for *C*_*P*_ is thus:

(14) $$C_{P}\left(F,\;W,\;\phi_{F}\right)=\text{min}\left(\omega_{C}\left(F,\;W\right),\phi_{Z}\left(F,\;W\right)\omega_{X}\left(F,\;W,\;\phi_{F}\right)\right)$$

All the function and parameter definitions of the final model can be found in Tables 1 and 2, respectively. Finally, although not necessary for the derivation of the model, it is useful to get an expression for the threshold elemental ratio (TER)—the particular value of ϕ_*F*_ at which an individual switches from carbon limitation to mineral limitation. It can be found by solving for the value of ϕ_*F*_ at which $\omega_{C}\left(F,\;W\right)=\phi_{Z}\left(F,\;W\right)\;\omega_{X}\left(F,\;W,\;\phi_{F}\right)$:

(15) $$\phi_{F}^{*}\left(F,\;W\right)=\frac{\sigma_{X}I\left(F,\;W\right)}{\frac{\omega_{C}\left(F,\;W\right)}{\phi_{Z}\left(F,\;W\right)}+M_{X}\left(W\right)}$$

**Table 2:** Parameter definitions and values for *Daphnia*

Parameter	Description	Value
γ_*CG*_	Fraction of carbon allocated to growth ending up in tissues	0.31
γ_*CR*_	Fraction of carbon allocated to reproduction ending up in tissues	0.63
γ_*XG*_	Fraction of mineral allocated to growth ending up in tissues	1
γ_*XR*_	Fraction of mineral allocated to reproduction ending up in tissues	1
σ_*C*_	Assimilation efficiency of carbon	0.53
σ_*X*_	Assimilation efficiency of the mineral	0.97
ϕ_*B*_	C:P ratio of the soma	100
ϕ_*F*_	C:P ratio in the food	Variable
*F*	Food concentration	83.33 μmol C/L
*W*_*b*_	Mass at birth	0.09 μmol C
*W*_*j*_	Mass at maturity	0.49 μmol C
ν	Mass‐specific maximum ingestion rate	2.5 d^−1^
*F*_*h*_	Half‐saturation constant	13.66 μmol C/L
*m*_*C*_	Mass‐specific maintenance rate for carbon	0.08 d^−1^
*m*_*X*_	Mass‐specific maintenance rate for the mineral	0.03 d^−1^
*r*	Parameter in the allocation function	5.1 μmol C^−1^

The derivations presented above allow to directly obtain the multiple nutrient equivalent of a net‐production model. In the previous section, we have made the connection between our model and net‐assimilation models explicit for the case of carbon limitation. In the case of mineral limitation, there is an additional subtlety, because allocation needs to be specified as a function of the quantity of carbon assimilates *actually used* by the organism, which is smaller than the total quantity acquired. The quantity of carbon assimilates used by the organism always equals *C*_*P*_ + *M*_*C*_, so that $\theta =\frac{\kappa \left(C_{P}+M_{C}\right)-M_{C}}{C_{P}}$. Replacing for θ in Equation (13) gives the value of *C*_*P*_ under mineral limitation:

(16) $$C_{P}=\frac{\phi_{B}\omega_{X}+M_{C}\left(1-\kappa \right)\left(\frac{\gamma_{\mathrm{CG}}}{\gamma_{\mathrm{XG}}}-\frac{\gamma_{\mathrm{CR}}}{\gamma_{\mathrm{XR}}}\right)}{\kappa \frac{\gamma_{\mathrm{CG}}}{\gamma_{\mathrm{XG}}}+\left(1-\kappa \right)\frac{\gamma_{\mathrm{CR}}}{\gamma_{\mathrm{XR}}}}$$

### 2.3 Model parameterization for *Daphnia*

**Figure 2:**
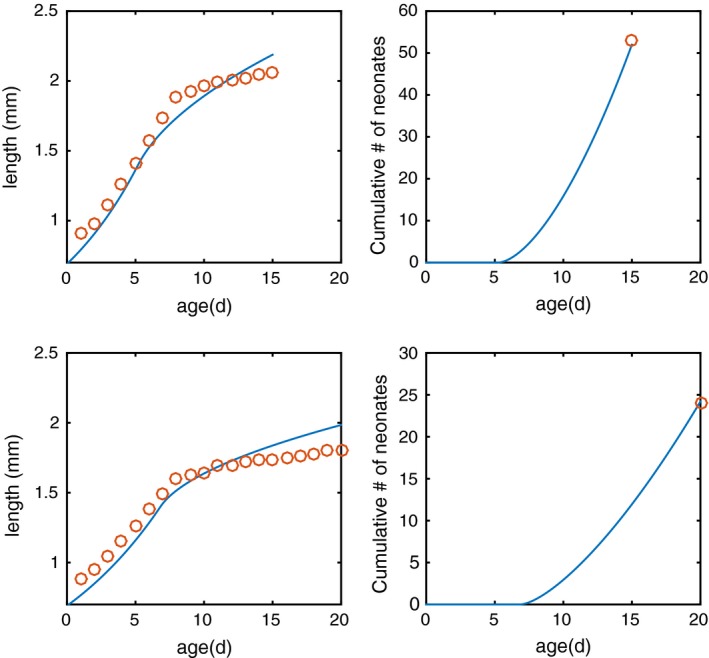
Empirical data (orange dots) and model predictions (blue lines) for *Daphnia pulex* growth and fecundity under conditions of carbon limitation (ϕ_*F*_ = 140; top two figures) and phosphorus limitation (ϕ_*F*_ = 750; bottom two figures)

To test the ability of our modelling framework to predict individual patterns of growth and fecundity under different conditions of nutrient limitation, we used the results of an experiment from Jeyasingh and Weider (2005) on *Daphnia pulex*. These data comprise detailed growth curves under carbon‐ and phosphorus‐limiting conditions (ϕ_*F*_ = 140 and ϕ_*F*_ = 750, respectively), as well as the cumulative fecundity at the end of the fifth adult moult (Figure 2). The nutrient denoted *X* in the previous section is thus phosphorus here. In these experiments, individuals were fed with high quantities of food, 83.33 μmol C L^−1^ d^−1^ (= 1 mg C/L/d). We assume that this results in constant food conditions of *F* = 83.33 μmol C/L.

There is an extensive literature on the physiological ecology of *Daphnia pulex* (e.g. McCauley, Murdoch, Nisbet, & Gurney, 1990; Nisbet et al., 2004); we could therefore parameterize most parts of the model from the literature. We did however estimate three parameters from the data using nonlinear regressions. These parameters are the assimilation efficiencies of C and P, because they may largely depend on experimental conditions (DeMott et al., 1998; McCauley, Nisbet, De Roos, Murdoch, & Gurney, 1996). The other parameter is γ_*CG*_. Although some values have been reported for daphnids, its interpretation is model‐dependent and unknown for the present model.

Masses are expressed in moles. We used the allometric relationship *W* = 0.22 L^2.4^ in order to convert length measurements of individuals (in mm) to carbon mass (in μmol C; Nisbet et al., 2004). Individuals are born with a mean length of 0.69 mm (Jeyasingh & Weider, 2005) and start allocating to reproduction at a length of 1.4 mm (Nisbet et al., 2004).

#### 2.3.1 Ingestion and assimilation

The ingestion rate of a *Daphnia* is well described by a type II functional response and can be expressed as (*F*/(*F* + *F*_*h*_)) *I*_*m*_ (*W*), where *F*_*h*_ is the half‐saturation constant, and *I*_*m*_ (*W*) reflects the size dependence of ingestion rate. We set *F*_*h*_ to a value of 13.66 μmol C/L (McCauley et al., 1990). We parameterized the function *I*_*m*_ (*W*) based on an experiment by Lynch, Weider, and Lampert (1986), in which ingestion rate scales approximatively in proportion to individual weight, that is, *I*_*m*_ (*W*) = ν*W* with a mass‐specific maximum ingestion rate ν of 2.5 d^−1^.

From the growth and reproduction data, we estimated assimilation efficiencies of σ_*C*_ = 0.53 and σ_*X*_ = 0.97, which is well within the range of values commonly reported (e.g. DeMott et al., 1998; He & Wang, 2007; McCauley et al., 1996).

#### 2.3.2 Maintenance

For maintenance rates, we assume that phosphorus is only required to make up for cell turnover and the production of the carapace. Assuming that, at any time, a constant fraction of the cells in the animal body is being replaced, this implies that the consumption rate of phosphorus is proportional to the mass of that nutrient in the animal body. We thus get $M_{X}\left(W\right)=m_{X}\frac{W}{\phi_{B}}$. We used the value of 0.01 d^−1^ for the fraction of total body phosphorus that is lost per day, which corresponds to the excretion rate of phosphorus under phosphorus‐limiting conditions (He & Wang, 2007). In addition, phosphorus is also lost because individuals moult. Assuming that the carapace makes up about 5% of body weight, has the same composition and that individuals moult on average every 2.5 days (Anderson et al., 2005), this results in an extra 0.02 d^−1^ consumption of phosphorus. This then gives a value of *m*_*X*_ = 0.03 d^−1^. For carbon, we assume that, in addition to these processes, some of it is used to provide energy for the organism, at a rate also proportional to body mass. The resulting function is hence *M*_*C*_ (*W*) = *m*_*C*_ *W*. We use a value of *m*_*C*_ = 0.08 d^−1^ (Nisbet et al., 2004).

#### 2.3.3 Allocation

For allocation, we use an empirically derived function from Nisbet et al. (2004), in which all the carbon acquired in excess to maintenance goes to growth in juveniles (i.e. θ = 1), whereas in adults, θ is a decreasing function of individual carbon mass: $\theta \left(W\right)=\frac{1}{1+r(W-W_{j})}$. We use the value of *r* = 5.1 μmol C^−1^ reported in Nisbet et al. (2004). We also tried a set of different values for this parameter, but this did not significantly improve model fit (not shown).

#### 2.3.4 Body composition and overheads costs

There is some variation in reported estimates of body C:P ratios in daphnid species, and evidence that it may be affected by various ecological factors, especially food composition (e.g. He & Wang, 2008). Nevertheless, those values usually range from 70 to 150, and we settle for a value of ϕ_*B*_ = 100.

Given our assumption that carbon is used both for energetic purposes and biomass production, whereas phosphorus is used for structural purposes only (although some of it is still required to compensate for cell turnover; Anderson & Hessen, 2005), there is no phosphorus overhead costs of growth and reproduction. Therefore, γ_*XG*_ and γ_*XR*_ are set to 1.

The fraction of carbon allocated to reproduction ending up in the production of neonate tissues, γ_*CR*_, is determined by both the carbon cost for producing an egg and the provisioning of the egg with energy to ensure embryogenesis. For the former, we assume that little metabolic work is involved in converting assimilates to eggs and assume that 95% of the carbon allocated to reproduction results in egg tissues (Kooijman, 2010; p.48). For the latter, it is generally found in daphnids that eggs are 25%–100% richer in carbon than somatic tissues (Baudouin & Ravera, 1972; Færøvig & Hessen, 2003; Sterner & Schulz, 1998; Ventura & Catalan, 2005), implying that such a quantity of carbon is consumed during embryogenesis. Here, we settle for a value of 50%. This implies that 2/3 of the carbon initially contained in the egg eventually results in neonate biomass. Combining both these fractions results in a value γ_*CR*_ of 0.63. This value is strikingly close to those reported in the literature and estimated by other means (Glazier, 1991; Nisbet et al., 2004).

The value of γ_*CG*_ = 0.31 that we estimate from the growth and reproduction data through nonlinear regressions is in comparison much lower. This is a rather puzzling result because the overhead cost of reproduction involves both the production of new somatic tissues and some extra maintenance costs of already developed tissues during embryogenesis. We might thus expect γ_*CR*_ to be smaller than γ_*CG*_. We come back more extensively to this point in the discussion, but we consider it important to stress here already that this value is not especially odd when one considers the possibility of extra carbon consumption in the juvenile relative to the adult stages. In particular, maturation costs and maturity maintenance (Kooijman, 2010), which are not accounted for here, are likely to explain the low estimate of γ_*CG*_.

## 3 RESULTS

Figure 2 shows how model predictions compare to the data, for both growth and fecundity. Although the model does not capture the deceleration of growth at later ages very well, there is an overall good agreement between the model predictions and the data.

**Figure 3:**
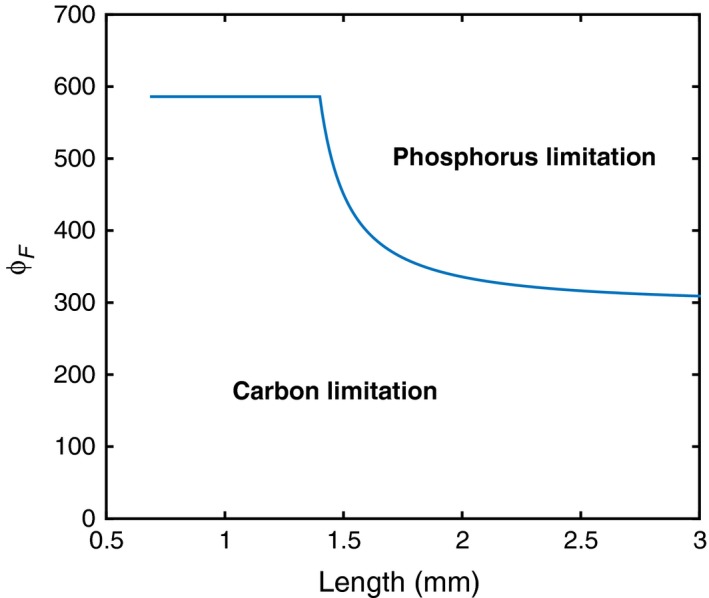
Threshold elemental ratio (TER) as a function of individual length, at a food concentration *F* = 83.33 μmol C/L. For juveniles (L < 1.4 mm), the TER does not depend on size, whereas it decreases with size in the adult stages (L ≥  1.4 mm, as individuals allocate more resources towards egg production

Using the parameterized model enables us to make predictions on how the TER varies throughout ontogeny (Figure 3). Most notably, there is a striking difference between the TER of juveniles and adults. In the juvenile stages, the TER has a high value of ϕ_*F*_ = 586, making the juveniles prone to carbon limitation. Past maturity, the TER quickly decreases to reach values close to 300 for lengths of 1.6 mm or greater. This pattern occurs because the production of somatic biomass is associated with a high carbon consumption due to the high overhead carbon cost and therefore low value of γ_*CG*_ (0.31). Biomass production through reproduction is, comparatively, less costly and γ_*CR*_ higher (0.63). Most importantly, for ϕ_*F*_ values between 300 and 600, the type of limitation changes during development (Figure 3).

**Figure 4:**
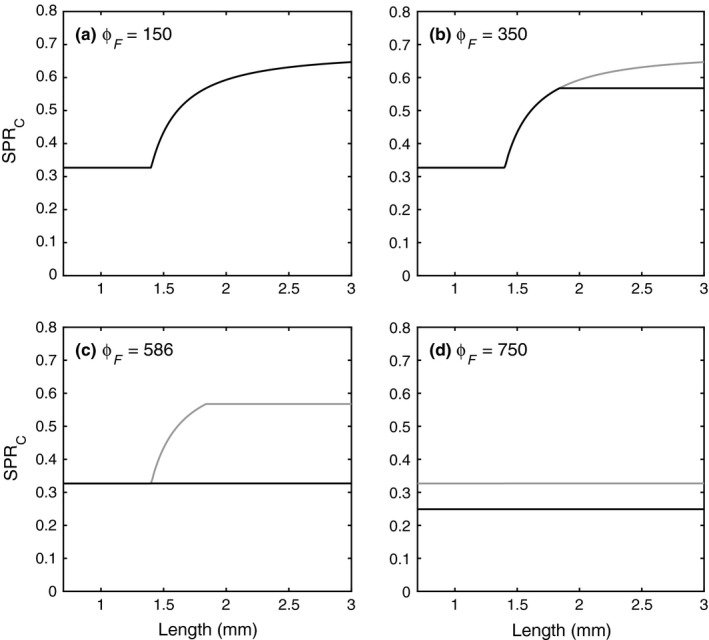
Mass‐specific production rate of carbon (SPR_C_) as a function of individual length for different values of ϕ_*F*_. The black lines give the shape of the function at the given value of ϕ_*F*_, and the grey lines indicate its shape at the previously considered lower value of ϕ_*F*_. Food concentration is *F* = 83.33 μmol C/L

Figure 4 illustrates how variation in the food C:P ratio affects the rate of biomass production of differently sized individuals. When ϕ_*F*_ is low, all individuals are carbon‐limited and the mass‐specific production rate of carbon (SPR_C_), and thus new biomass, by large individuals is about twice as large as that of small individuals because of the high value of γ_*CR*_ compared to γ_*CG*_ (Figure 4a). Phosphorus limitation starts appearing for ϕ_*F*_ values exceeding 300. Figure 4b illustrates what happens at ϕ_*F*_ = 350. Only the biggest individuals (about >1.8 mm) are then phosphorus‐limited, which causes a decrease in their SPR_C_. Smaller individuals remain carbon‐limited, and their SPR_C_ remains unaffected. Further increases in ϕ_*F*_ decrease the production of biomass by phosphorus‐limited adults even more and also decrease the threshold size at which individuals switch from carbon limitation to phosphorus limitation. All juveniles, independently of their size, become phosphorus‐limited for ϕ_*F*_ values >586, at which point the SPR_C_ of all individuals becomes equal (Figure 4c). This contrasts with the scenario of pure carbon limitation in which adults were much more productive than juveniles. Further increases in ϕ_*F*_ decrease the production rate of differently sized individuals to the same extent (Figure 4d).

## 4 DISCUSSION

### 4.1 A general modelling framework

We have presented a general modelling framework that accounts for the various nutrient requirements an organism may encounter over ontogeny. It produces models that are relatively simple, easy to analyse and require only relatively limited data for their parameterization. The framework is sufficiently flexible to derive the multiple nutrient equivalent of many of the bioenergetic models encountered in the literature such as net‐production and net‐assimilation models (De Roos & Persson, 2013; Jager et al., 2013; Lika & Nisbet, 2000; Nisbet et al., 2004). In comparison with purely energy‐ or carbon‐based models, the additional components required are the assimilation efficiency, specific maintenance rate and overhead costs associated with the second nutrient, as well as the composition of the body (respectively, σ_*X*_, *m*_*X*_, γ_*XG*_, γ_*XR*_ and ϕ_*B*_ in our model). Although they may not always be available for some organisms, there are many ways of estimating approximate values for them. For example, one can use values of related species or consider the physiological basis underlying them (e.g. *m*_*X*_ may be approximated from excretion rates as mineral overhead costs can often be set to zero). The energy overhead costs of somatic and neonate production also appear essential in driving the relative nutrient needs over ontogeny. Although these parameters are related to carbon dynamics, they are rarely explicitly accounted for in energy‐based models. For example, overheads costs of somatic production are often not explicitly modelled but accounted for as a reduction in assimilation efficiency or an increase in metabolic maintenance rate (e.g. De Roos & Persson, 2013; McCauley et al., 1990; Yodzis & Innes, 1992).

The model is also easy to extend to include more details on the organism's biology. It is, for example, easy to relax the assumption that body composition is constant through development or does not depend on food composition by making ϕ_*B*_ an appropriate function of *W* or ϕ_*F*_, with very few additional changes in the model. If body composition depends on size, only the expression for ϕ_*Z*_ (*F*,* W*) changes structurally. This occurs because the value of ϕ_*B*_ appearing in Equations (11) and (12) corresponds to the body composition of the adult and the body composition of the neonates, respectively, and this gives a slightly different expression for ϕ_*Z*_ (*F*,* W*). If body composition depends on ϕ_*F*_, none of the equations change structurally, but the TER becomes harder to solve, as ϕ_*F*_ would also appear in the right hand side of Equation (15). This would generally preclude any closed‐form solution, but solving for the TER would still be possible using a numerical procedure.

We did not deal explicitly with scenarios in which inputs from feeding are insufficient to cover maintenance. As our model does not explicitly model nutrient reserves, there are only a limited number of ways this could be dealt with. An almost certain consequence is that growth and reproduction will stop. What happens after that really depends on what processes are included in the maintenance functions *M*_*C*_ (*W*) and *M*_*X*_ (*W*), and what are the consequences for the organism when these costs are not met. For example, in our *Daphnia* model, we assumed that phosphorus was used to compensate for cell turnover, whereas carbon was used for both cell turnover and energy provisioning to the organism. If phosphorus acquisition is not sufficient to compensate for cell turnover, the individual will not only loose phosphorus but also carbon because each (non‐renewed) cell contains both. The rate of loss is then determined by the difference between the rate of phosphorus assimilation and the basal cell turnover rate. In terms of our model equations, ω_*X*_ (*F*,* W*, ϕ_*F*_) becomes negative, and *C*_*P*_ (*F*,* W*, ϕ_*F*_) = ω_*X*_ (*F*,* W*, ϕ_*F*_)/ϕ_*B*_. Nutrient losses from the body may result in shrinking body size or increased mortality, depending on the type of organism considered. We leave open the question as to how mortality may be related to the rate of nutrient loss. There are a number of additional scenarios that could be considered, but they are likely to be species‐specific, and we leave the task for further model developments along these lines to the interested reader.

A potential response to food quality that our model does not currently incorporate is compensatory feeding (Berner, Blanckenhorn, & Körner, 2005; Fink & Von Elert, 2006; Raubenheimer & Simpson, 1993). Compensatory feeding is a behaviour in which the organism increases its feeding rate in response to poor food quality. It occurs only in case the mineral is limiting, which should therefore not affect the TER, but only the production rates under conditions of mineral limitation. In theory, this could be incorporated into our model by making the ingestion rate a function of ϕ_*F*_ and including extra metabolic costs for increased feeding effort. The difficulty lies in specifying the ingestion function, because it then varies as a function of the individual's own demand, which greatly complicates the model. For example, studies that tackled the issue of modelling this behaviour approached it as an optimization problem, in which the individual maximizes the rate at which it produces biomass (Darchambeau, 2005; Mitra & Flynn, 2007; Suzuki‐Ohno, Kawata, & Urabe, 2012).

Many reviews in nutritional ecology advocate a greater synthesis between ecological stoichiometry and the geometric framework of nutritional ecology (Raubenheimer, Simpson, & Mayntz, 2009; Wilder & Jeyasingh, 2016). The latter framework focuses on macronutrients (carbohydrates, proteins and lipids) rather than elements, but likewise recognizes that multiple currencies limit individual production. It however accounts for individual‐level processes in much more details than ecological stoichiometry does. Our model could be useful in linking ecological stoichiometry and the geometric framework as, similar to multivariate DEB models (Kuijper, Anderson, & Kooijman, 2004), it accounts for multiple nutrients, be it micro‐ or macronutrients, and it explicitly represents individual life history, in particular development, reproduction and mortality, which are key in determining population dynamics and ecological interactions. Our model's relative simplicity may offer a way to integrate species‐specific details more easily than other DEB approaches. Finally, one of the greatest added value of our modelling framework may lie in its ability to connect individual nutritional ecology to the powerful insights on population and community ecology provided by the theory of physiologically structured population dynamics (De Roos & Persson, 2013; Metz & Diekmann, 1986). This body of theory has been largely developed using single‐nutrient net‐production and net‐assimilation models, and the close connection of our modelling framework to these models provides an opportunity to connect these bodies of theory more closely, as discussed more extensively below.

### 4.2 The *Daphnia* model

Regarding our parameterization for *Daphnia*, we already mentioned the rather puzzling finding that γ_*CG*_ is much smaller than γ_*CR*_, and hinted that another process may be at play to cause larger carbon overheads in juveniles relative to adults. We suspect such a process to be linked to the cost of maturing and maturity maintenance. In the DEB framework, a large quantity of energy is allocated to the maturation process during the juvenile stages (Kooijman, 2010), and juveniles mature into adults once the total amount of energy allocated to this purpose reaches a specific value. This allocation to maturation is however considered to only increase the (physiological) complexity of the individual but not its biomass. For daphnids, it is usually estimated that about 40% of the energy mobilized is dissipated in the maturation process (Kooijman, 2010; Martin, Jager, Nisbet, Preuss, & Grimm, 2013). In the adult stages, the same fraction of mobilized energy translates into the production of egg biomass and, ultimately, neonate production. Some of that energy also disappears because of the overhead costs of egg production and energy consumption during the embryogenetic stage, but not all of it. This implies that biomass production in the juvenile stage comes with greater overhead costs than in the adult stage. We did not explicitly model the maturation process, which implies that these costs are effectively reflected in the low estimated value of γ_*CG*_. Whether or not the maturation process is modelled explicitly, the overarching conclusion remains that juvenile carbon requirements are much higher than adults’, as the latter mostly reproduce, and this should produce a TER function that has a similar shape to the one reported here.

Explicitly accounting for the maturation process in our framework would require a number of additional model assumptions for which there is no experimental foundation. For example, accounting for an explicit maturity index raises the question of what elemental composition is required for the build‐up of this index. In the DEB framework, it is assumed that this elemental composition is the same as the investments in growth and reproduction, but this assumption is not based on experimental data. Furthermore, assumptions would be required about how the maturity index affects the carbon and nutrient maintenance costs of the individual. Finally, in a net‐production setting it would be impossible to distinguish experimentally between the loss of nutrients associated with maturation vs. the overhead costs of growth, mainly because a maturity index such as in the DEB framework has no physiological manifestation. It is likely that adding a maturation scheme to our *Daphnia* model may improve the model fit when compared to data, especially because it makes it possible to account for variation in the size at maturity. The inflection of the growth curve in Figure 4a,c indeed suggests that the onset of reproductive allocation occurs at different sizes under the two experimental conditions. Nonetheless, because of the difficulties associated with the inclusion of a maturation scheme discussed above and the primary purpose of our *Daphnia* model, which is to illustrate the general framework in a simple way, we chose to avoid including extra complexity and speculations, and maintained the assumption that individuals mature at a given size, in line with other works (e.g. Jager et al., 2013; Kooijman & Metz, 1984).

### 4.3 Implications of ontogeny for patterns of nutrient limitations

A primary result of our model analysis is that the TER becomes size‐dependent. Individuals in different states of development may thus be limited by different nutrients. Our *Daphnia* model reported a strong dependence of the TER on individual size because the overhead carbon cost of growth was much higher than that of reproduction. At this stage, it is unclear whether this is a rather general result or whether it is more specific to *Daphnia*, but it remains true that any difference between the overhead costs of growth and reproduction is likely to generate size dependence in the TER function. More generally, our formula for the TER reveals that many typical features of organisms’ functioning may generate size dependence in the TER. Our formula for the TER looks similar to published ones (e.g. Anderson et al., 2005; Frost et al., 2006) and is the product of the same basic physiological processes (assimilation and maintenance rates, body composition, overhead costs). A key distinction lies however in the inclusion of size dependence in these processes. Size dependence originates from two different sources. First, the ingestion and maintenance rates may be size‐dependent functions. In our *Daphnia* model, this scaling does not affect the TER, because both these rates are assumed to scale in proportionally to individual weight. This would however change if a different scaling was assumed, which is generally the case for other species. For example, ingestion rates usually scale with mass with an exponent smaller than one (Kooijman, 2010; West, Brown, & Enquist, 2001). Including these scalings would then results in a TER function that depends on size in a continuous fashion. The second source of size dependence in the TER originates from the function ϕ_*Z*_ (*F*,* W*), which reflects that the nutrient requirements to produce one unit of biomass may also vary, because biomass is produced either through somatic growth or offspring production. Changes in body composition across development would also fall in this latter category.

The concept of TER is fundamental to understand how biomass production responds to variation in nutrient supply (Frost et al., 2006; Sterner & Elser, 2002). Many studies have stressed the importance of interspecific differences in nutrient requirements to explain ecological processes (Elser et al., 2000; Frost et al., 2006). For example, Frost et al. (2006) reported a coefficient of variation of 147% among the C:P TER of 41 aquatic taxa. Our parameterization of the *Daphnia* model suggests that the intraspecific differences resulting from juvenile/adult differences are of the same order of magnitude, with a difference of up to twofold. This range of values comprises the typical food conditions experienced by *Daphnia* in natural conditions (Elser et al., 2000; Ventura & Catalan, 2005). This large intraspecific difference in TER implies that there is a wide range of environmental conditions under which the different individuals making up a population are limited by different nutrients. Therefore, although biomass production is always limited by only one nutrient at the individual level, co‐limitation of biomass production by several nutrients may emerge at the population level. Various hypotheses have been advanced to explain patterns of co‐limitation at the community level (Danger, Daufresne, Lucas, Pissard, & Lacroix, 2008; Harpole et al., 2011), but as far as we are aware, the possibility that individuals in different states of development may be limited by different nutrients has never been considered. More generally, the conjecture that TERs may be size‐dependent opens the way for a large range of ecological consequences at the population and higher levels of organizations, and points to development as a fundamental feature in mediating populations’ response to food quality.

### 4.4 Implications for population dynamics

Another important implication of our model analysis is related to the contrasting effects of different types of nutrient limitation for size‐dependent patterns of biomass production. Recent research has identified relative differences in mass‐specific biomass production rates, often referred to as ontogenetic asymmetry, as a major determinant of population and community dynamics (De Roos & Persson, 2013; De Roos, Persson, & McCauley, 2003). Classical population dynamics theory is based on unstructured population models, and corresponds to the limiting case where the mass‐specific biomass production rate and mortality rate of individuals do not vary with their size (De Roos, Metz, & Persson, 2013). In contrast, when there is ontogenetic asymmetry, a variety of novel dynamical behaviours may occur, including various types of population cycles, alternative dynamical attractors or biomass overcompensation in response to increased mortality (reviewed in De Roos & Persson, 2013; De Roos et al., 2003).

Our parameterized *Daphnia* model predicts ontogenetic symmetry under phosphorus limitation but (strong) ontogenetic asymmetry under conditions of carbon limitation. Current theory has addressed how ontogenetic asymmetry emerged from size dependence in various ecological and metabolic rates and from different lifestyles. Here, we highlight a new possibility linked to the fact that different metabolic activities, such as growth and reproduction, have different nutrient and energetic requirements. Food quality may vary over space, in which case differences in population dynamical behaviour across environments may be related to the alternative patterns of ontogenetic asymmetry produced by different types of nutrient limitation. Alternatively, food quality may also vary over time and patterns of ontogenetic asymmetry may therefore vary in a dynamic way. This opens exciting possibilities for future research on the dynamics of populations and communities as the implications of variable patterns of ontogenetic asymmetry have so far never been investigated.

## AUTHORS’ CONTRIBUTIONS

R.R. and A.M.R. conceived the ideas and designed methodology; R.R. implemented and analysed the model; R.R. led the writing of the manuscript; and A.M.R. contributed critically to its development.

## DATA ACCESSIBILITY

All data and models used in this study are presented in the article.
