# Supplementary material for: The impact of development on patterns of nutrient limitation
Source: Funct Ecol. 2018 Apr 16;32(6):1507–19. doi: 10.1111/1365-2435.13101 (PMC6049933; doi:10.1111/1365-2435.13101)
Supplement: Supplementary file 1 [file FEC-32-1507-s001.pdf]

Functional Ecology

BRITISH ECOLOGICAL SOCIETY

The impact of development on organisms' response to food quality

Romain Richard & André M. de Roos

Biological tissues are made up from many different nutrients and individual organisms constantly face the challenge to acquire all of them in the right proportion in order to be able to grow and reproduce. Most ecological theory is based on the assumption that biological production is limited by the total energetic content of the food acquired through feeding. But the scientific community is becoming aware that biological production may often be limited by the quality of the food ingested, i.e. its nutrient composition, rather than its quantity. Natural environments differ widely regarding the composition of the food available to organisms that inhabit them, and this composition may also vary substantially over time. The integration of such food quality aspects into existing theory has led to major progress in our understanding of how species interact with each other, how natural populations grow or decline, and what determines nutrient flux across ecosystems.

Individuals grow and develop throughout their lives, and this usually comes with a number of physiological and behavioral changes. As a consequence, individuals may require different nutrients in different proportions at different stages of their lives. For example, juvenile individuals use a large part of the nutrients they ingest to support their own development, whereas adults may use them to reproduce. Current ecological theory lacks a general

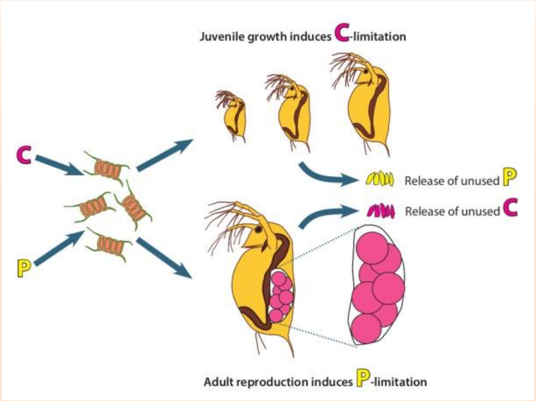

Photo provided by authors

framework, accessible to both theoreticians and empiricists, that can be used to assess how development affects an organism's response to food quality. We aim to fill that gap by providing such a framework. We show that many typical features of organisms' functioning can change which nutrient limits individual production the most. Since natural populations usually contain individuals in various stages of development, this means that population growth can be limited simultaneously by several nutrients, which points to development as a key determinant of population and community responses to food quality.
